# Renal Leiomyosarcoma With Regional Lymph Node Metastasis and Biopsy Tract Seeding: A Case Report

**DOI:** 10.7759/cureus.88880

**Published:** 2025-07-28

**Authors:** Nassar M Alqurashi, Ibrahim M Salman, Ahmed K Yamani, Ahmed E Aloufi, Mohammed T Aldawsari

**Affiliations:** 1 Urology, Alhada Armed Forces Hospital, Al Hada, SAU

**Keywords:** adult renal tumors, biopsy tract seeding, kidney malignancy, malignant renal tumor, renal leiomyosarcoma, renal sarcoma, soft tissue sarcoma

## Abstract

Renal leiomyosarcoma is a rare and aggressive tumor that comprises a very small proportion of adult kidney malignancies. Its clinical and imaging characteristics can closely resemble those of other high-grade renal tumors, which complicates the diagnostic process. We report the case of a 66-year-old male patient who presented with a large left renal mass and was diagnosed with high-grade pleomorphic leiomyosarcoma. He underwent radical nephrectomy along with en bloc removal of a para-aortic lymph node, which was histologically confirmed to contain metastatic disease. In an unusual postoperative development, the patient presented with a subcutaneous mass at the prior biopsy site. This was confirmed to be tumor seeding along the biopsy tract, an infrequent occurrence in renal sarcomas.

## Introduction

Renal sarcomas are quite rare, making up only about 1-2% of all malignant kidney tumors in adults [1,2]. These cancers develop from the connective tissue parts of the kidney and can be tricky to diagnose because their symptoms and imaging often resemble other kidney cancers, especially sarcomatoid renal cell carcinoma (RCC) [2,3]. It is really important to tell these two apart since their treatment plans and outlooks differ significantly.

Among renal sarcomas, leiomyosarcoma is the most common type, accounting for roughly half to more than half of cases [4,5]. These tumors arise from the smooth muscle cells found in areas like the kidney’s capsule, pelvis, or blood vessels [5,6]. Patients typically experience vague symptoms such as abdominal pain or notice a lump in the abdomen [7]. While computed tomography (CT) or angiography can detect these tumors, they often cannot clearly distinguish leiomyosarcoma from other renal tumours [2,7].

The definitive diagnosis depends on histopathological examination and using immunohistochemistry (special staining techniques) to confirm it is leiomyosarcoma rather than another spindle cell tumor or sarcomatoid RCC [8,9]. Given the typically aggressive behavior of these tumors, early and complete surgical excision offers the best opportunity for cure [1,10,11].

Even with surgery, renal leiomyosarcomas often have high potential for local recurrence and distant metastasis [12]. While chemotherapy has been tried for these soft tissue sarcomas, its success has been limited so far [13]. That’s why a team approach with ongoing close follow-up is essential to give patients the best outcomes possible [14,15].

## Case presentation

**Figure 1:**
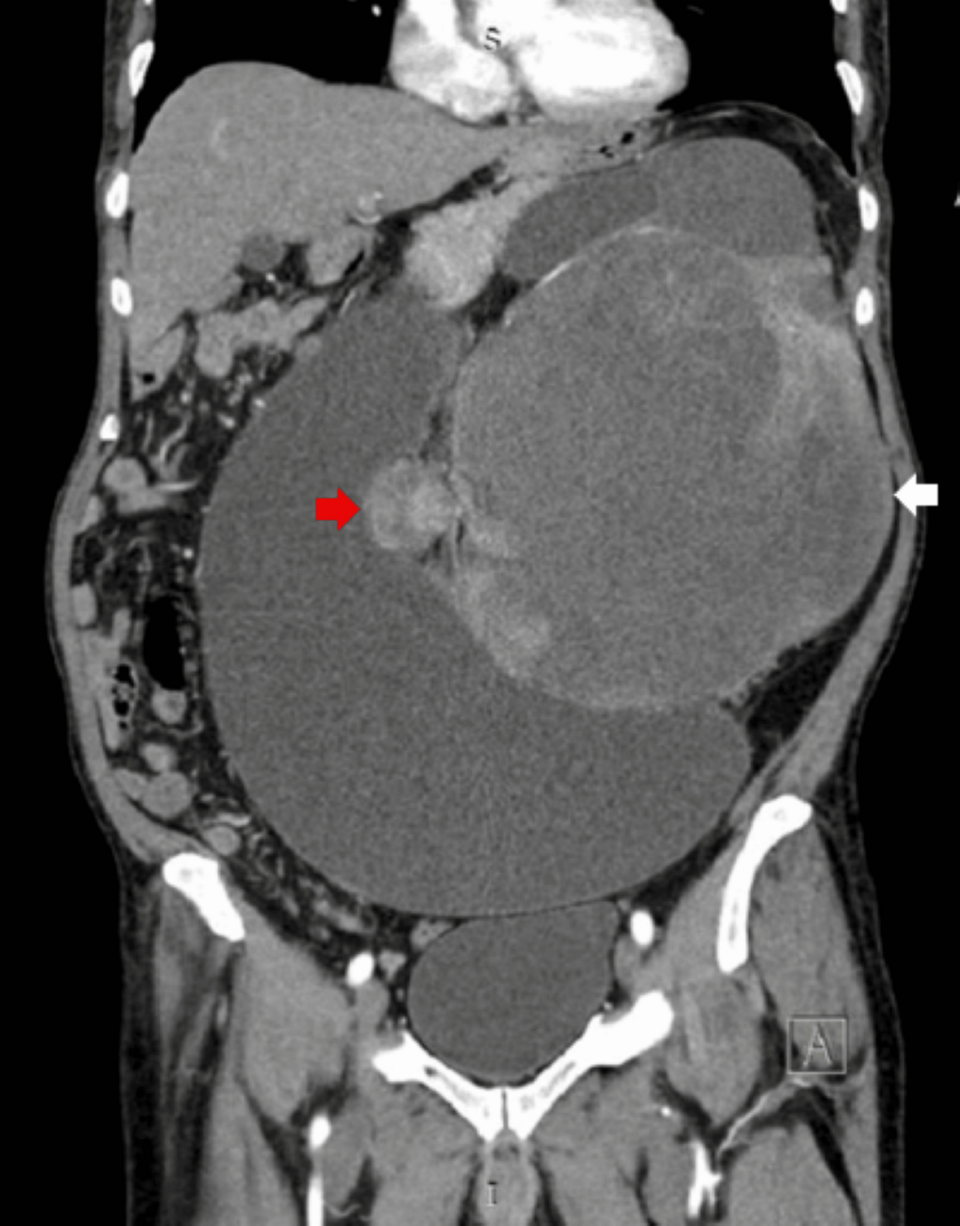
Contrast-enhanced CT (coronal view) of the abdomen demonstrating a large, heterogeneous mass replacing the left kidney. The mass demonstrates a peripherally enhancing soft tissue component with a large central area of necrosis (white arrow). Rounded, enhancing soft tissue lesion with central necrosis, measuring 4.5 × 4 cm, is seen anterior to the aorta below the origin of the renal arteries, exhibiting similar characteristics to the primary mass (red arrow)

**Figure 2:**
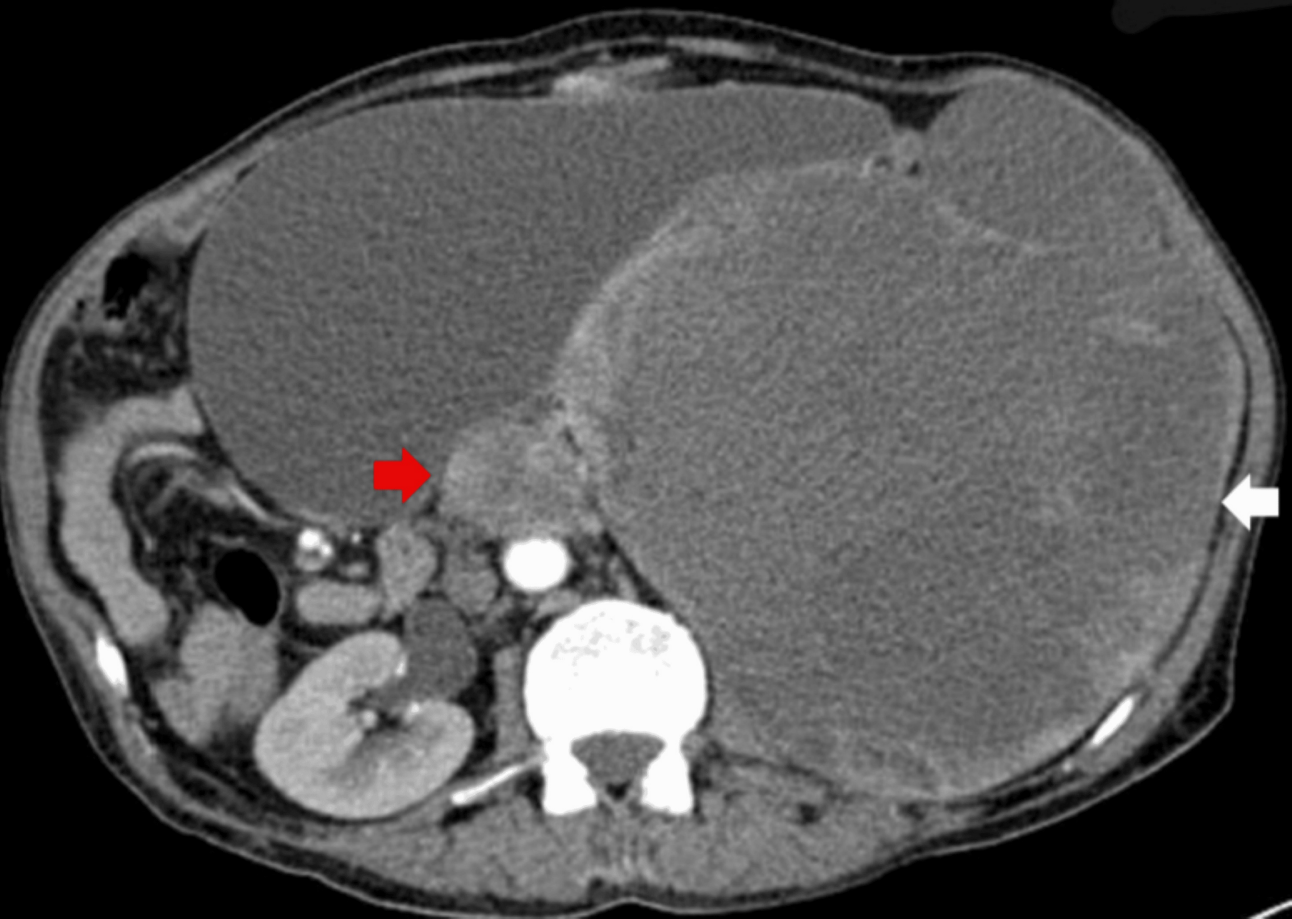
Contrast-enhanced CT (Axial view) at the level of the upper abdomen showing heterogeneous left renal mass. The mass demonstrates a peripherally enhancing soft tissue component with a large central area of necrosis (white arrow). Rounded enhancing soft tissue lesion with central necrosis, measuring 4.5 × 4 cm, is seen anterior to the aorta below the origin of the renal arteries, exhibiting similar characteristics to the primary mass (red arrow)

A 66-year-old male patient without significant medical history was admitted with a three-month history of vague, generalized abdominal pain and a left-sided abdominal lump. On examination, there was an approximately 30 × 20 cm mass occupying the left lumbar region, extending down to the pelvis and crossing over to the right side of the abdomen. Contrast-enhanced CT of the abdomen revealed a large heterogeneous mass replacing the left kidney. The mass appeared as a peripherally enhancing soft tissue component with a large central area of necrosis and a focus of central calcification. It measured 19 × 18 × 23 cm in anteroposterior, transverse, and craniocaudal dimensions, respectively, displacing the spleen and stomach superiorly and the pancreas superolaterally to the right. Additionally, a rounded enhancing soft tissue lesion with central necrosis, measuring 4.5 × 4 cm, was seen anterior to the aorta below the origin of the renal arteries, exhibiting similar characteristics to the primary mass (Figures 1, 2). A staging CT of the chest was also performed, which showed no evidence of pulmonary metastases.

**Figure 3:**
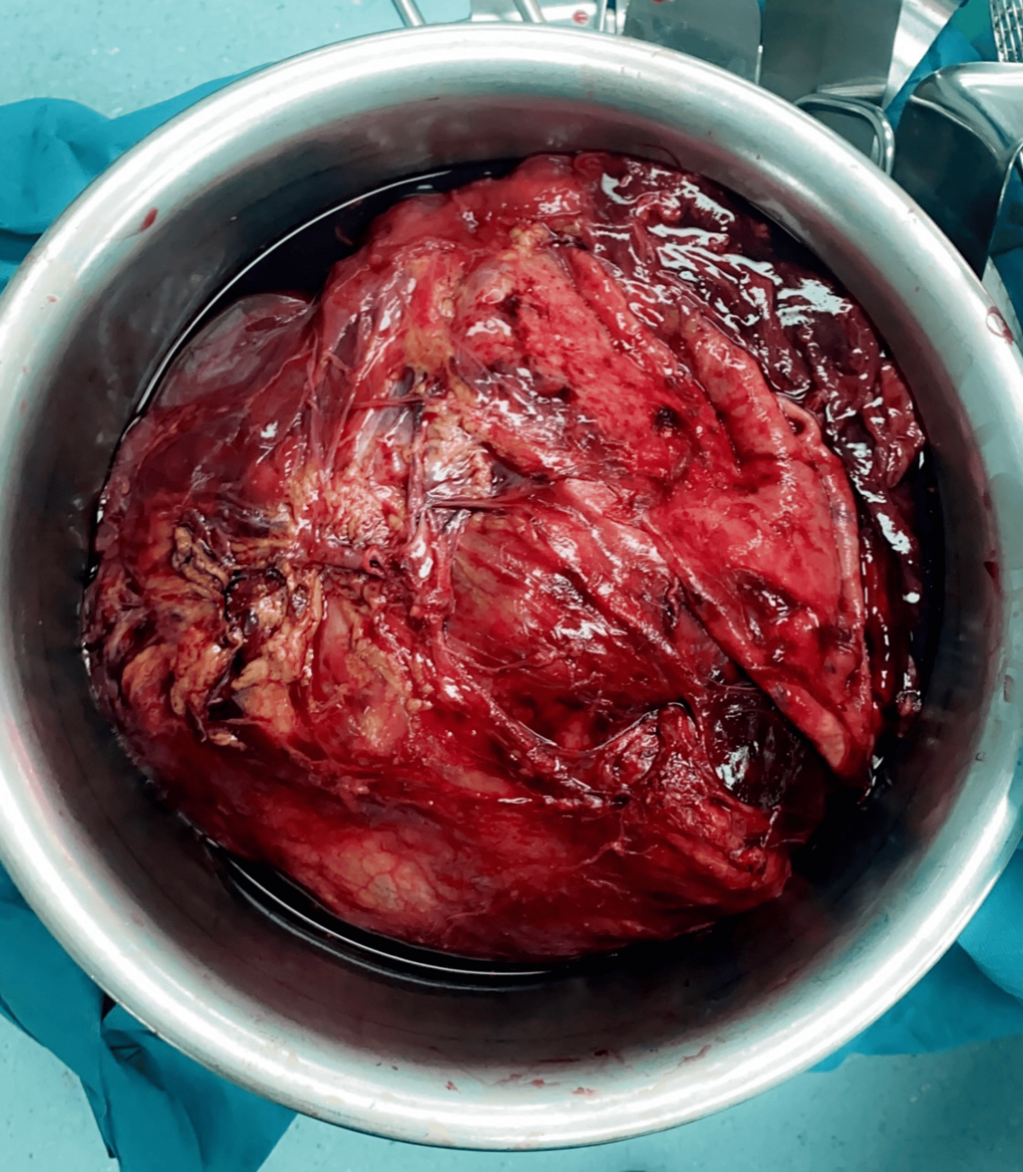
Gross specimen of the resected left renal mass following radical nephrectomy.

Based on the radiological findings, an ultrasound-guided core needle biopsy was performed, which revealed pleomorphic leiomyosarcoma. Surgical excision was planned with the intent to completely remove the mass. A midline laparotomy incision was made, followed by mobilization of the left colon. A large left renal mass with cystic components was identified, with no attachments to surrounding structures or adjacent organs. A left radical nephrectomy and excision of a lymph node adherent to the anterior surface of the aorta were performed (Figure 3). Both specimens were sent for histopathological examination. The postoperative course was uneventful, and the patient was discharged on postoperative day 5.

Histopathological analysis of both specimens revealed high-grade leiomyosarcoma with extensive coagulative tumor necrosis and high mitotic activity. Immunohistochemistry showed intermediate positivity for smooth muscle actin (SMA), and strong positivity for CD10 and vimentin.

**Figure 4:**
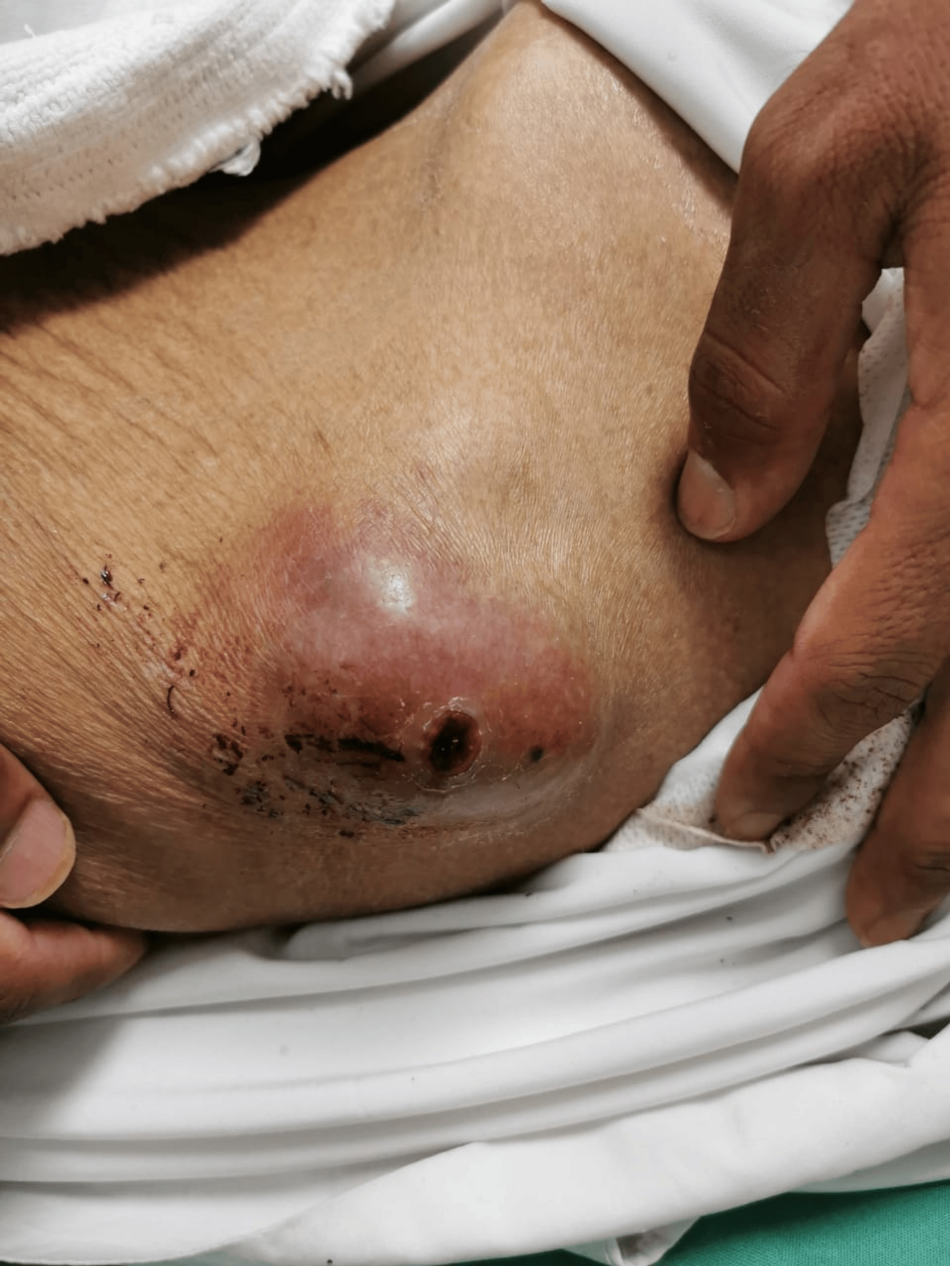
Clinical image demonstrating a firm, tender 7 × 4 cm subcutaneous mass with minimal serous discharge at the prior biopsy site, suggestive of tumor seeding along the needle tract.

Ten days later, the patient presented to the emergency department with a painful abdominal swelling at the left lumbar region, corresponding to the previous ultrasound-guided biopsy site. The swelling had started four days prior to presentation and had progressively increased in size, accompanied by minimal serous discharge. On physical examination, a 7 × 4 cm tender, hard subcutaneous swelling was noted at the biopsy site, with minimal serous discharge (Figure 4). The midline surgical incision appeared clean and partially healed.

**Figure 5:**
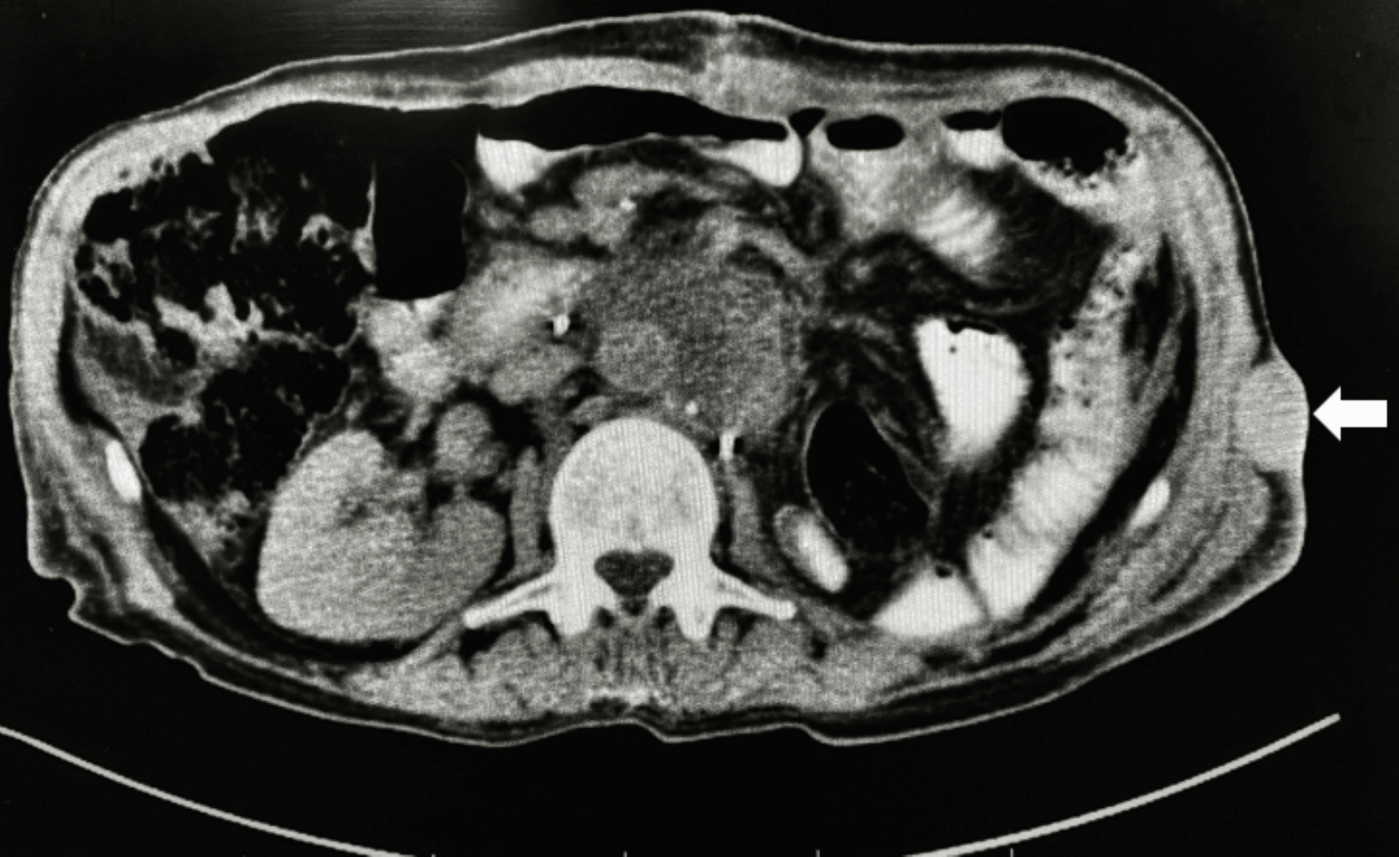
Follow-up enhanced CT scan of the abdomen and pelvis reveals a subcutaneous, homogeneously enhancing, rounded mass.

Enhanced CT scan of the abdomen and pelvis revealed a subcutaneous, homogeneously enhancing, rounded mass (Figure 5).

The patient underwent surgical excision of the mass, and the specimen was sent for histopathological examination, which revealed a high-grade leiomyosarcoma. The patient was discharged home on postoperative day 1. The case was planned to be discussed at our regional tumor board; however, the patient subsequently presented to the emergency department with deterioration of general condition and decreased oral intake. The patient was admitted under the oncology service for supportive care, deferring any role for chemotherapy.

## Discussion

Renal leiomyosarcoma is an extremely rare entity, accounting for approximately 0.12% of all malignant renal tumors [7]. Among urological sarcomas, the most common sites in descending order of incidence are the paratesticular region, prostate/seminal vesicles, bladder, and kidney. Although rare, renal sarcomas tend to be more aggressive and lethal compared to sarcomas arising from other genitourinary sites [2].

Clinically, patients with renal leiomyosarcoma may present with flank or abdominal pain, a progressively enlarging palpable mass, and hematuria. A retrospective review of 10 cases by Ozturk reported that 40% of patients were asymptomatic, while 60% presented with symptoms, with lumbar pain being the most common presenting complaint [8]. Leiomyosarcoma comprises 50-60% of all renal sarcomas [2,4,13] and arises from smooth muscle cells of the renal capsule or perinephric structures. There is a female predominance, and most patients are diagnosed between the fourth and sixth decades of life [1,5,6,8].

Diagnosing renal sarcoma clinically remains challenging, as it can mimic sarcomatoid RCC). Features suggestive of sarcoma include a clear origin from the capsule or perisinuous region, growth to large size without evidence of lymphadenopathy, and a hypovascular pattern on angiography [3]. Definitive diagnosis requires meticulous morphological evaluation and immunohistochemical analysis, as renal leiomyosarcoma and sarcomatoid RCC share overlapping histopathological features such as spindle-shaped atypical cells [10,14].

A hallmark of sarcomas is their potential to extend into surrounding tissues, complicating surgical resection [2]. Magnetic resonance imaging (MRI) is valuable in preoperative planning by delineating tissue planes and vital structures. Radical nephrectomy remains the primary treatment modality, with most reported cases undergoing this procedure [5,8]. Wide en bloc excision is critical for large tumors displacing adjacent organs, as it offers the best chance for long-term cure [15].

The most important prognostic factors in renal sarcomas are tumor grade and surgical margin status [4]. The majority of renal leiomyosarcomas reported in the literature are high-grade tumors, which frequently metastasize, particularly to the lungs, resulting in poor prognosis [2,4,6]. Local recurrence is common and often requires repeat excision to prolong survival.

In a case series by Wang et al. that included 41 patients, overall survival rates at one, three, and five years were 86%, 40%, and 14.5%, respectively [4]. Similarly, Kendal reported a five-year overall survival of 25% and a cause-specific survival of 60% in a series of 12 patients; tumor stage and patient age were major determinants of survival [7].

Chemotherapeutic agents such as doxorubicin and ifosfamide have traditionally been used in the adjuvant setting, with newer regimens including docetaxel and gemcitabine showing some efficacy [16]. Given the poor prognosis, a multimodal approach involving surgery, chemotherapy, and radiotherapy is often employed when the patient's condition permits.

## Conclusions

Renal leiomyosarcoma is an extremely rare malignancy with a challenging clinical diagnosis due to its overlap with sarcomatoid renal cell carcinoma and its inherently poor prognosis. Accurate diagnosis requires thorough histopathological evaluation and immunohistochemical analysis. Radical nephrectomy with en bloc resection of involved adjacent structures remains the cornerstone of management. Given the aggressive nature of the disease, a multimodal treatment approach, including surgery, chemotherapy, and radiotherapy, is often employed when clinically appropriate.
